# Diagnostic and Prognostic Value of Circulating Tumor Cells in Head and Neck Squamous Cell Carcinoma: a systematic review and meta-analysis

**DOI:** 10.1038/srep20210

**Published:** 2016-02-02

**Authors:** Xiang-Lei Wu, Qian Tu, Gilbert Faure, Patrice Gallet, Chantal Kohler, Marcelo De Carvalho Bittencourt

**Affiliations:** 1 Department of Otolaryngology - Head and Neck surgery, Zhongnan Hospital of Wuhan University, China; 2 SBS Department, CRAN, UMR 7039 CNRS, University of Lorraine, France; 3 Laboratory of Immunology, Nancytomique platform, CHU of Nancy, France; 4 Department of Otolaryngology and Cervico-facial Surgery, CHU of Nancy, France

## Abstract

Several techniques have been developed to detect circulating tumor cells (CTC) in patients with head and neck squamous cell carcinoma (HNSCC), but their diagnostic and prognostic value are not yet fully established. A computerized retrieval of literatures was conducted without time restrictions using the electronic database in December 2014. Diagnostic accuracy variables were pooled and analyzed by the Meta-DiSc software. Engauge Digitizer and Stata software were used for pooled survival analysis. Twenty-two retrieved studies were eligible for systematic review, of which 9 conformed for the diagnostic test meta-analysis and 5 for the prognostic analysis. Subgroup analysis showed 24.6% pooled sensitivity and 100% pooled specificity of detections by using positive selection strategy, which moreover presented low heterogeneity. The presence of CTC was significantly associated with shorter disease free survival (DFS, HR 4.62, 95% CI 2.51–8.52). In conclusion, current evidence identifies the CTC detection assay as an extremely specific, but low sensitive test in HNSCC. Also, the presence of CTC indicates a worse DFS.

In cancer diseases, tumor cells eventually detach from the primary site and disseminate in the blood, the lymphatic fluid, the bone marrow or even in the cerebrospinal fluid. Circulating tumor cells (CTC) designate mainly the ones circulating in the peripheral blood. Under certain circumstances, like immune escape or immunoediting, these cells can establish a new tumor in distance sites, a process known as metastasis^1^. Increasing evidences suggest that CTC are released long before the development of metastasis, sometimes even before the formation of apparent primary tumors^2^,^3^. A considerable number of studies aimed to develop a reliable CTC-detection technique have been conducted in recent years. Several researchers believe that information derived from CTC might play a “liquid biopsy” role in clinical assessment of patients with cancer^4^,^5^. Indeed, CTC was validated as a prognostic factor of metastatic breast, colorectal, and prostate cancer^6^,^7^,^8^. Therefore, CTC could also potentially represent an ideal diagnostic and prognostic biomarker in the head and neck squamous cell carcinoma (HNSCC).

However, compared with aforementioned entities, the clinical value of CTC in HNSCC, the sixth leading cancer by incidence worldwide^9^, has not been summarized yet. The overall survival of patient with HNSCC is relatively low due to the high rate of regional and distant metastases at diagnosis. The five-year survival rate for all stages combined is approximately 40–50%. There is still a risk of local recurrence in 20% of cases even if the resection margins are negative in anatomopathological analysis. The disseminated tumor cells are therefore generally suspected to be responsible for this minimal residual disease through a micro-metastasis pathway^10^,^11^. For the same reason, previous studies also hoped to overcome the deficiency of current TNM staging system by detecting the presence of CTC.

As studies concerning CTC assays in HNSCC accumulate, it becomes increasingly important to evaluate all of the relevant studies in order to develop a more reliable overall and also quantitative assessment. In the present study, we conducted a systematic review and meta-analysis to pool together the available evidence of CTC evaluation in patients with HNSCC, related to its diagnostic and prognostic value. We hope this approach will facilitate the understanding of present discrepancy, and also contribute to reveal the eventual translational utility of this test in HNSCC clinical management.

## Materials and Methods

### Literature search

A computerized retrieval of literature was conducted without time restrictions using the electronic database in December 2014. Information sources include Pubmed (contains MEDLINE and PreMEDLINE), Google Scholar, EMBASE, Scopus and Web of Science. The search strategy involved the use of keywords “circulating tumor cells”, “epithelial cells”, and “rare cells” variably combined with one of the following terms: “head and neck”, “squamous cell carcinoma”, “HNSCC”, “Nose”, “nasal sinus”, “oral cavity”, “oropharynx”, “nasopharynx”, “hypopharynx”, “laryngopharynx”, “larynx”, “trachea”, “oral”, “oropharyngeal”, “nasopharyngeal”, “laryngopharyngeal”, “laryngeal”, “cervical tracheal”, and “cervical esophagus”.

### Eligibility Criteria

#### Inclusion criteria

1) original research published in a peer-reviewed journal; 2) only English language papers were included; 3) to avoid ambiguity in the definition of CTC, only techniques using peripheral blood but not bone marrow or other body fluids of patients were considered eligible; 4) patients with histologically confirmed squamous cell carcinoma originating from sphere of head and neck (i.e. oral cavity, nose, ear, pharynx, larynx, cervical trachea and cervical esophagus); 5) clinical stage of disease provided; 6) clear information about techniques used for CTC tests: strategy of enrichment and identification, targeted markers, sample volume, etc…; 7) only studies that included negative controls were eligible for the meta-analysis of diagnostic test; 8) hazard ratios (HR) with 95% confidence intervals (CI) or at least Kaplan-Meier curves for survival analysis should be noted in the reports for inclusion in the meta-analysis of prognostic value.

#### Exclusion criteria

1) other sources of data, i.e. letters to the editor, communications, meetings abstracts or reviews were excluded; 2) studies involving merely spiking-experiments, animal models, or other pathological types of cancer were not included; 3) publications including patients populations already included in previous papers; 4) studies with a poor size of sample, defined here as ≤10 patients enrolled, were excluded for meta-analysis.

### Data extraction

The searched articles were sorted and managed by the software EndNote X7 (Thomson Reuters, USA). Two independent investigators have evaluated the eligibility of studies by the reading sequence of title, abstract, and fulltext. The following data were recorded from the identified articles: title, first author, journal, year of publication, features of patients (i.e. sample size, clinical stage, distribution of carcinoma, source of negative controls), timing of detection (baseline, ongoing, or post treatment), details of techniques (including blood sample volume, method of enrichment and identification, target markers), accuracy of *in vitro* test (sensitivity and specificity, if measured). In case where survival analysis was evaluated, data of clinical outcomes (disease-free survival, DFS; progression-free survival, PFS; overall survival, OS) were also exported. Of note, only the results of baseline detection were used if serial blood samples were collected.

### Statistical Analysis

Diagnostic accuracy variables [i.e. sensitivity (Sen.), specificity (Spe.), likelihood ratios (LR+ and LR–), diagnostic odds ratios (DOR) and area under curve (AUC) of the receiver operating characteristic curve (ROC)] were pooled and analyzed by using the Meta-DiSc software, version 1.4^12^. The sensitivity was defined as the proportion of patients with positive CTC detection (CTC+) among all patients diagnosed with HNSCC. The specificity was defined as the proportion of control subjects with negative CTC detection (CTC–) among all subjects without HNSCC. Positive likelihood ratios (LR+), which was calculated as *Sen./(1-Spe.)*, and negative likelihood ratios (LR-), which was calculated as *(1-Sen.)/Spe.*, express how much more frequent the respective result is among subjects with disease than among subjects without disease. DOR, which is calculated as *LR + /LR*–, indicates how much greater the odds of having the HNSCC are for the subject with a CTC+ than for the subject with a CTC–. The AUC is computed by numeric integration of the curve equation by the trapezoidal method. If any study had a table with a zero value in any cell, the solution used in our study was adding 0.5 to all cells. The threshold effect was not evaluated since all included studies stratified the results as negative/absence and positive/quantification, which eliminated the potential variations. Publication bias was evaluated only when there were at least 10 studies included in the meta-analysis^13^.

The Stata v.12.0 software was used to estimate the pooled HR and 95% CI for DFS, PFS, and OS. For studies that had not shown the corresponding results, the Engauge Digitizer v.4.1 software was used to extract survival data from the Kaplan-Meier curves^14^,^15^. In the case where a study stratified the survival by different CTC thresholds, we reorganized the data as CTC+ vs. CTC– and then compared it with other studies according to methods used in other meta-analysis^16^,^17^. An observed HR >1 implied a worse survival for patients positive for CTC detection.

The association between the presence of CTC and clinical features of patients were assessed by visual inspection because of unreported values and/or an obvious trend. Between-study heterogeneity was assessed by the inconsistency index (I^2^). For interpreting I^2^, values of 25%, 50%, and 75% were assigned as low, moderate, and high heterogeneity as suggested by Higgins *et al*.^18^. Because of small number of studies involved and anticipated inter-study heterogeneity, a random effects model^19^ was applied in all pooled calculations performed to provide more conservative estimates.

## Results

### Study characteristics

**Figure 1:**
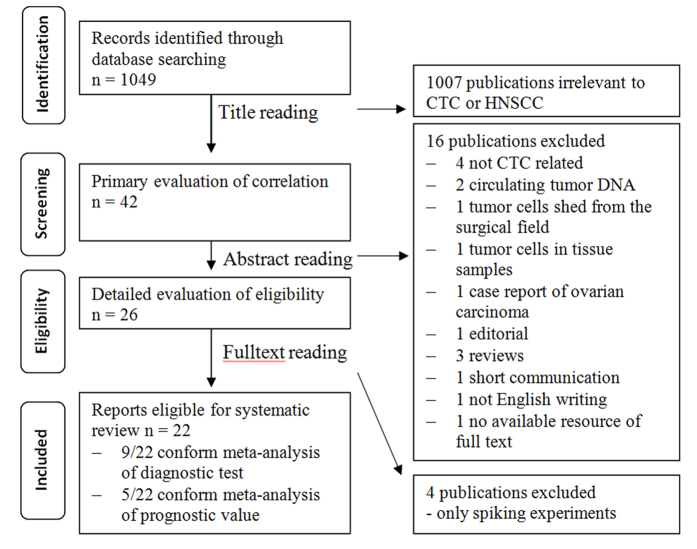
Flow diagram of the strategy used for the selection of reports. Flow diagram created according to PRISMA guidelines.

**Figure 2:**
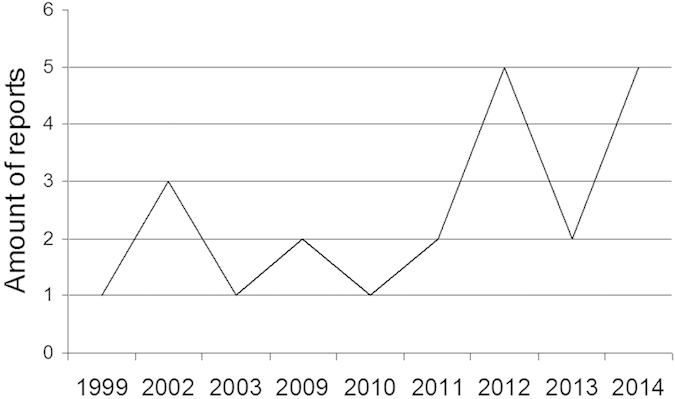
Amount of reports presented in chronological order. The amount of publications eligible for systematic review is graphically illustrated.

As shown in Fig. 1 [drawn according to ref. 20], we have retrieved 22 studies eligible for systematic review^21^,^22^,^23^,^24^,^25^,^26^,^27^,^28^,^29^,^30^,^31^,^32^,^33^,^34^,^35^,^36^,^37^,^38^,^39^,^40^,^41^,^42^. The initial research on CTC detection in HNSCC was published in 1999^21^ and the amount of subsequent publications is graphically illustrated in Fig. 2. The general descriptions and calculated sensitivity/specificity (if not indicated in the study) of CTC assays of these studies are summarized in Supplementary Table S1.

The overall amount of patients involved in these studies was 857 and the mean sample size was 39 (range 7–144). In these 22 studies, 6 (27.3%) exclusively focused on locally advanced stage (clinical stage III-IV)^22^,^23^,^24^,^25^,^26^,^27^. The proportion of patients with clinical stage I-III was calculated as 36.5% (313 out of 857) and the one with stage IV was 60.8% (521 out of 857). With regard to the set-up of negative controls, only 11 reports recruited healthy donors and the mean number was 19 (range 3–61)^21^,^22^,^25^,^26^,^27^,^28^,^29^,^30^,^31^,^32^,^34^.

Except for one report without detailed description^35^, the others indicated that HNSCC principally originated from the oral cavity (313 cases, 36.5%) and oropharynx (163 cases, 19.0%), followed by nasopharynx (124 cases, 14.5%), hypopharynx (72 cases, 8.4%), and larynx (62 cases, 7.2%).

**Figure 3:**
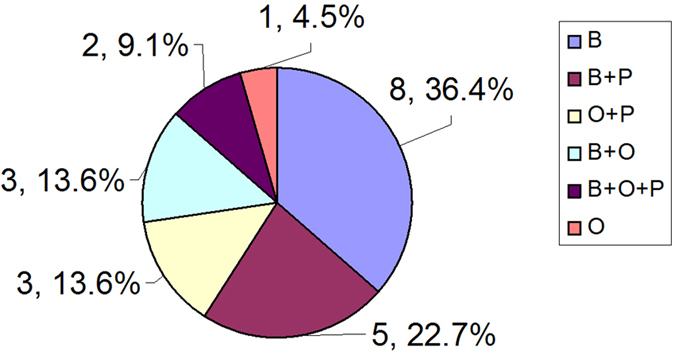
Distribution of studies according to timing of detection. Results presented as “number of reports, percentage”. Timing of detection: B = baseline; O = ongoing; P = post-treatment.

We have classified the timing of detection as baseline (before all treatment), ongoing (during surgery, chemotherapy or radiotherapy) and post-treatment (end of treatment). As shown in Fig. 3, most studies detected CTC at baseline level (8 of 22 studies, 36.4%) or compared it with post-treatment level (5 out of 22 studies, 22.7%).

### Techniques of CTC detection

The blood sample used for detection varied in volume between 2–30 ml. A volume of 7.5ml was most frequently taken (10 out of 22 studies, 45.5%).

The technique of CTC enrichment included two strategies: marker-independent and -dependent. The former one was applied in 7 studies^21^,^22^,^24^,^28^,^29^,^38^,^41^, which involved the erythrocyte lysis with/without density gradient centrifugation. The latter one was used in the remaining 15 studies^23^,^25^,^26^,^27^,^30^,^31^,^32^,^33^,^34^,^35^,^36^,^37^,^39^,^40^,^42^, which applied the immunomagnetic cell separation (positive immunomagnetic separation, PIMS or negative immunomagnetic separation, NIMS). During this step, epithelial cell adhesion molecule (EpCAM) was used as a marker for 7 PIMS cases^23^,^26^,^30^,^32^,^37^,^39^,^42^ and CD45 was the one for all 8 NIMS cases^25^,^27^,^31^,^33^,^34^,^35^,^36^,^40^. Moreover, CellSearch^®^ the only clinically validated, FDA-cleared blood test for enumerating CTC, was used in 71.4% (5/7) PIMS cases. Although the reagents employed for NIMS cases were variable, the overall enrichment process was essentially similar. Actually, the centrifugation techniques were also used as a necessary procedure for PIMS, like erythrocyte lysis for NIMS.

CTC enumerated were identified also by two ways in these 22 studies: 68.1% (15/22) by direct evidence (morphological methods, i.e. immunocytochemistry, CellSearch^®^ system based on fluorescence optics, flow cytometry) and 31.9% (7/22) by indirect evidence (i.e. RT-PCR).

### Meta-analysis of diagnostic accuracy

**Figure 4:**
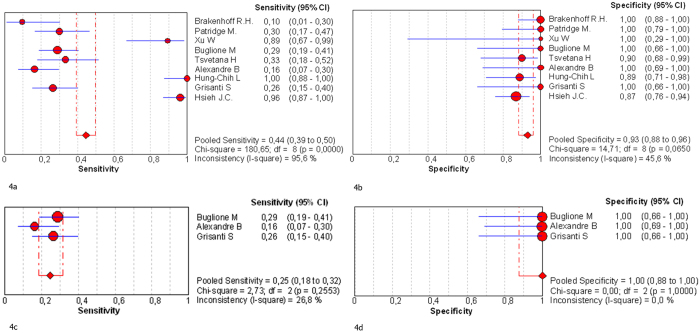
Diagnostic accuracy forest plots (**a**) Forest plots of overall sensitivity (**b**) Forest plots of overall specificity (**c**) Forest plots showing sensitivity of PIMS subgroup (**d**) Forest plots showing specificity of PIMS subgroup. Forest plots illustrated by Meta-DiSc software. Studies are identified by names of the first author. PIMS, positive immunomagnetic separation.

Due to the absence of negative control group or little sample size, 13 out of 22 studies were excluded of meta-analysis of diagnostic accuracy. The 9 remaining studies eligible^21^,^25^,^26^,^27^,^28^,^29^,^30^,^32^,^34^ were pooled for the meta-analysis of diagnostic test (grey backgrounds in Supplementary Table S1). As presented in Fig. 4, the overall Sen. and Spe. of pooled studies was 44.4% (0.393–0.497, 95% CI; I^2^ = 95.6%) and 92.9% (0.882–0.962, 95% CI; I^2^ = 45.6%), respectively.

Since significant heterogeneity was observed for the 9 studies, subgroup analysis was done according to different variates: PIMS or NIMS as enrichment method; direct or indirect evidence as identification method; blood sample volume ≤ or >7.5ml; various thresholds of target markers (2, 3, or 4). However, only the subgroup categorized by enrichment method, which included 3 studies^26^,^30^,^32^ using CellSearch^®^ technique has presented low heterogeneity (I^2^ = 26.8% for Sen. and I^2^ = 0% for Spe.). In this case, the pooled Sen. and Spe. was 24.6% and 100%, respectively (Fig. 4). The other measures of diagnostic accuracy (i.e. LR+, LR–, DOR, and AUC) for overall studies and the PIMS subgroup are summarized in Table 1.

### Meta-analysis of prognostic value

In total, five reports with calculated survival data or available Kaplan-Meier curves were included for meta-analysis of prognostic value. Of these studies, 2 analyzed both the DFS and OS^33^,^42^, 2 evaluated both PFS and OS^32^,^34^ and 1^40^ just assessed the DFS. Since one study^32^ stratified the survival by cut-off value of CTC as 3 subgroups (enumeration = 0, 1, or 2), we have extracted and reorganized the data as CTC+ vs. CTC–. The recalculated HR was 1.88 (1.21–2.91, 95% CI) for PFS and 2.04 (1.29–3.22, 95% CI) for OS.

**Figure 5:**
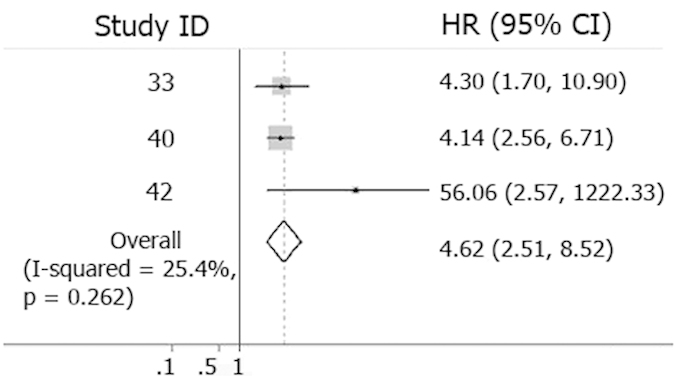
Forest plots showing prognostic value of CTC detection on DFS. ■: Individual HR, designed proportionally to the amount of patients included; **- - -**: Combined HR of the studied subgroup through meta-analysis; ◊: The centre of the diamond gives the combined HR and its extremities the 95% CI. DFS, Disease Free Survival; Study ID, serial number of reference.

As shown in Fig. 5, 3 eligible studies were pooled into the DFS meta-analysis, which showed an overall HR 4.62 (2.51–8.52, 95% CI) with low heterogeneity (I^2^ = 25.4%). Follow-up period of these reports were quite similar, from 30 to 38 months.

Analysis performed in 2 studies concerning PFS and 3 studies concerning OS showed no significant HR but a high heterogeneity was detectable (PFS: HR = 1.32, 0.71–2.44, 95% CI, I^2^ = 87.5%; OS: HR = 1.63, 0.85–3.13, 95% CI, I^2^ = 86.6%).

### Association of CTC detection with clinico-pathological features of patients

Available in 10 studies, multiple clinico-pathological characteristics were evaluated related with CTC detection. The results are summarized in Table 2. If the threshold of significant *p*-value was set as <0.01, only one study suggested that metastasis stage (M of TNM staging system) was significantly associated with detectable CTC. Also, in the case the *p* value was set as <0.05, one study showed a significant association of CTC detection with each of following features: tumor size, nodal involvement, and lung nodules infiltration. Taking into account poor evidence and unavailable original data, these studies therefore cannot lead to any confirmative finding.

## Discussion

Since the first observation of CTC in 1869 in a patient with metastatic cancer^43^, its critical role in the spread of carcinomas has been demonstrated over the succeeding one hundreds years^44^. However, the diagnostic or prognostic potential of CTC has only been exploited in the last decade^1^,^7^,^45^,^46^,^47^. After the development of new technologies, especially the application of CellSearch^®^ system, researchers showed more ambitions on this test. Recently, a meta-analysis including 8 studies, which aimed to evaluate the predictive role of CTC in HNSCC has found that the recurrence/metastasis rate in the CTC-positive patients was significantly higher compared to patients without disease progression, suggesting that the detection of CTC in patients with squamous cell carcinoma of the head and neck has a predictive value for tumor progression^48^. However, the diagnostic role of CTC was not evaluated in this study and the effect of CTC on survival time could not be analyzed due to incomplete data in the included studies. Furthermore, studies with disseminated tumor cells (DTC) in the bone marrow were also included in the meta-analysis. These discrepancies may help to explain the different overall conclusions between our study (a decreased DFS when CTC were present) and Wang’s meta-analysis concerning only the prognostic value of CTC detection in the HNSCC setting.

Most HNSCC arise in the oral cavity, pharynx and in the larynx, and patients often display signs and symptoms of locally advanced disease at the time of diagnosis^7^,^49^. Carcinoma originated from the head and neck region has a high propensity to metastasize from its rich lymphatic system^50^,^51^,^52^. The consequence of this process represents as a worse prognostic, such as a high incidence in recurrence^53^. These might be the epidemiological reasons for designing the recruitment of patients in present studies. Despite considerable efforts that have been made to relate positive CTC identification with clinico-pathological features, very poor evidence was found^23^,^25^,^27^,^28^,^30^,^32^,^33^,^36^,^40^,^41^. Of note, the TNM staging provides crucial information of tumor burden and an advanced stage generally expected to associate with more CTC^54^,^55^,^56^. However, such a trend has not been found in HNSCC or in some investigations done in colon^57^, prostate^58^ and lung cancer^59^,^60^.

Compared with certain individual studies with encouraging conclusions, our results of meta-analysis suggest that CTC detection in patients with HNSCC have limited sensitivity but extremely high specificity for diagnosis. This test therefore should not be used as screening or first-line diagnostic test but might have great interest for confirming suspicious cases or searching cancer of unknown primary site^61^. The significant differences among sensitivities of CTC detection most likely derived from technique factors more than other variables.

We have found that the three studies using CellSearch^®^ system had sensitivities almost at similar levels (16.33–28.77%), which were relatively lower than RT-PCR (10–96.22%) or immunocytochemistry (89.47–100%). The range of CTC enumeration detected by CellSearch^®^ was 1–5/7.5 ml of blood. To briefly explain its principles, EpCAM + cells were targeted immunomagnectically during the enrichment process and then the events with cytokeratins (CKs) 8+, 18+, 19+ 4′,6-Diamidino-2-Phenylindole (DAPI) + CD45- were identified as circulating tumor cells^7^,^62^,^63^. As a reliable epithelial cell marker, EpCAM was widely used in involved studies. The expression of EpCAM was detected in 98 of 131 tumor categories and this rate varies from 59% (oral cavity) to 100% (pharynx) in HNSCC^64^. Compared with their primary tumors, its expression was found to be more frequently lower than higher in metastases^65^, which strongly suggest a potential switch of epithelial–mesenchymal transition (EMT). Although the role of EMT in tumor metastasis remains controversial, several studies have indicated that expression of EpCAM and some of the CKs are lost from CTC during EMT^66^,^67^,^68^,^69^,^70^,^71^. Moreover, the pattern of CKs also presents regional differences in corresponding carcinomas^72^. Thus, the differential expression of EpCAM and CKs, the EMT process, and the utilization of various identification markers, could explain the heterogeneity in overall studies and the lower sensitivity of studies using positive immunomagnetic cell separation strategies.

The negative depletion enrichment, which is not an EpCAM-based capture, was also explored in HNSCC^25^,^27^,^34^. These non-EpCAM based essays hoped to overcome aforementioned inefficiency and indeed showed a relatively higher sensitivity (43–100%). However, regrettably they have not been taken into our meta-analysis due to poor sample size or no negative control group set-up.

On the other hand, we have conducted the meta-analysis of studies on prognostic factors to develop an overall assessment of CTC in clinical management of patients with HNSCC. We have found that detectable CTC was important for predicting a shorter DFS but was not associated with PFS or OS. Such difference would be more likely associated with the intrinsical differences among survival end points. There are different ways of evaluating survival and in fact no international consensus standards are given for DFS and other endpoints. Generally, DFS indicates the percentage of people in the trial who are alive and without cancer after a specified period. DFS is frequently employed where all identifiable tumor has been resected and can be served as an early indicator of improved survival^73^. Meanwhile, PFS is defined as the time elapsed between treatment initiation and metastatic tumor progression^74^, including but not limited to the patients that received curative treatment. With PFS therefore, researchers are mostly concerned with distant but not locoregional metastasis. In our meta-analysis, compared with DFS, more confounding factors were involved into PFS as well as OS and consequently weakened their correlations with the presence of CTC. Even though in these studies the diagnostic accuracy has not been evaluated due to a lack of negative control group, they did not present significant heterogeneity, as confirmed by the inconsistency index test.

Notably, in the present meta-analysis, we have extracted merely the data at baseline as the timing of the assessment. Indeed, too many confounders exist during treatment: different therapeutic options; transient dissemination of CTC during the surgical and invasive procedure^75^,^76^; destruction or conversion of CTC induced by chemotherapy^77^. The diagnostic accuracy and prognostic value of CTC therefore should only be interpreted for the initial evaluation of tumor burdens. Besides, the bias related to an aggregation of survival data should also be enlightened. The present results are based on either reported data through univariate survival analysis, or recalculated HR for subgroups, or extrapolation from the survival curves, hence making assumptions on the censoring process. Finally, the publication bias has not been evaluated because neither the meta-analysis of diagnostic accuracy nor prognosis involved more than 10 studies. Of 10 studies that reported survival analysis, 5 were excluded for the meta-analysis due to insufficient data and 3 represented negative association between detectable CTC and DFS. Thus, in regard to 3 positive versus 3 negative, a potential bias could not be completely ruled out.

In conclusion, current evidence identifies the CTC assay as an extremely specific, but low sensitive test in HNSCC. Presence of CTC indicates a worse DFS for patient with HNSCC, whatever the biological approaches used. In spite of potential bias, the role of CTC in clinical management should still be valued but also the interpretation needs to be done in conjunction with individual clinical information. This observation requires update by further investigation with a more rigorous experimental design, such as a negative control group set-up and a standardized format on survival analysis reporting.

## Additional Information

**How to cite this article**: Xiang-Lei, W.U. *et al*. Diagnostic and Prognostic Value of Circulating Tumor Cells in Head and Neck Squamous Cell Carcinoma: a systematic review and meta-analysis. *Sci. Rep.* **6**, 20210; doi: 10.1038/srep20210 (2016).

## Supplementary Material

Supplementary Information

## Figures and Tables

**Table 1:** Diagnostic measures of overall and subgroup.

**First author**		**LR + (95% CI)**	**LR– (95% CI)**	**DOR**	**AUC**
Overall					
Brakenhoff R.H.	6.82 (0.34–135.06)		0.90 (0.77–1.05)	7.56 (0.34–166.20)	
Patridge M.	10.37 (0.65–165.36)		0.72 (0.58–0.89)	14.47 (0.80–260.69)	
Xu W	7.00 (0.52–94.07)		0.14 (0.04–0.48)	49.00 (1.91–1258.70)	
Buglione M	5.81 (0.38–88.65)		0.75 (0.61–0.92)	7.78 (0.43–139.69)	
Tsvetana H	3.33 (0.82–13.52)		0.74 (0.56–0.98)	4.50 (0.88–22.98)	
Alexandre B	3.74 (0.23–60.07)		0.87 (0.73–1.04)	4.30 (0.23–80.67)	
Hung-Chih L	7.86 (2.95–20.97)		0.02 (0.01–0.34)	399.00 (19.63–8110.80)	
Grisanti S	5.37 (0.35–82.94)		0.77 (0.62–0.96)	6.98 (0.38–127.62)	
Hsieh J.C.	7.34 (3.84–14.03)		0.04 (0.01–0.17)	168.94 (34.23–833.81)	
pooled	6.65 (4.20–10.55)		0.55 (0.35–0.84)	19.98 (5.90–67.71)	
I^2^	0.00%		95.2%	51.6%	0.95
PIMS					
Buglione M		5.81 (0.38–88.65)	0.75 (0.61–0.92)	7.78 (0.43–139.69)	
Alexandre B		3.74 (0.23–60.07)	0.87 (0.73–1.04)	4.30 (0.23–80.67)	
Grisanti S		5.37 (0.35–82.94)	0.77 (0.62–0.96)	6.98 (0.38–127.62)	
pooled		4.90 (1.00–23.93)	0.80 (0.71–0.90)	6.18 (1.15–33.11)	0.97
I^2^		0.00%	0.00%	0.00%	

PIMS, positive immunomagnetic separations; LR+, positive likelihood ratios; LR–, negative likelihood ratios; DOR, diagnostic odds ratios; AUC, area under curve; I^2^, inconsistency index.

**Table 2:** Frequency of CTC and clinico-pathologic characteristics of patients.

**ID**	**T stage**	**N stage**	**M stage**	**Clinical stage**	**Others**
21	*p *=* ns*	*p *=* ns*	*p *=* ns*	N/S	*p* = 0.04 lung nodules
22	N/S	N/S	N/S	N/S	*p *=* ns* (tumor site, induction chemotherapy)
25	*p *=* ns*	*p *=* ns*	N/S	N/S	*p *=* ns* (gender, patient’s height and weight, performance status, ASA score, alcohol and tobacco consumption, tumor site, tumor differentiation, anemia and thrombopenia)
26	*p *=* ns*	*p *=* ns*	N/S	N/S	*p *=* ns* (tumor differentiation)
28	*p *=* ns*	*p *=* ns*	N/S	*p *=* ns*	*p *=* ns* (tumor site, tumor differentiation)
30	N/S	N/S	N/S	N/S	*p *=* ns* (age, sex, weight loss, smoking, alcohol, tumor differentiation, prior radiotherapy, prior chemotherapy, Argiris factors;)
31	*p *=* ns*	*p *=* ns*	*p *=* ns*	N/S	*p *=* ns* (age, smoking history, surgical margins, human papillomavirus status)
34	*p *=* ns*	N0–2a vs. N2b +, *p* = 0.013	N/S	N/S	*p *=* ns* (tumor volume, human papillomavirus status)
38	N/S	*p *=* ns*	N/S	*p *=* ns*	*p *=* ns* (age, sex, tumor site, adjuvant therapy, smoking, and alcohol)
39	*p* = 0.04	N/S	*p* = 0.004	N/S	N/S

*p* value extracted directly from reports.

ID = serial number of reference; ns = no significant; N/S = non specify; TNM and Clinical stage: according to AJCC staging system; ASA score, score according to American Society of Anaesthesiologists physical status classification system.
