# Supplementary material for: Diagnostic and Prognostic Value of Circulating Tumor Cells in Head and Neck Squamous Cell Carcinoma: a systematic review and meta-analysis
Source: Sci Rep. 2016 Feb 2;6:20210. doi: 10.1038/srep20210 (PMC4735798; doi:10.1038/srep20210)
Supplement: Supplementary Information [file srep20210-s1.doc]

**Article title**: Diagnostic and Prognostic Value of Circulating Tumor Cells in Head and Neck Squamous Cell Carcinoma: a systematic review and meta-analysis

**Author names and affiliation**: Xiang-Lei WU, MD, MSc^1, 2, 3^, Qian TU, MD, PhD^2,3^, Gilbert FAURE, MD, PhD^2, 3^, Patrice GALLET, MD^4^, Chantal KHOLER , MD, PhD^3^, Marcelo DE CARVALHO BITTENCOURT, MD, PhD^2, 3 *^

^1^: Department of Otolaryngology - Head and Neck surgery, Zhongnan Hospital of Wuhan University, China

^2^: SBS Department, CRAN, UMR 7039 CNRS, University of Lorraine, France.

^3^: Laboratory of Immunology, Nancytomique platform, CHU of Nancy, France.

^4^: Department of Otolaryngology and Cervico-facial Surgery, CHU of Nancy, France.

^*^ Corresponding author. Avenue du Morvan, 54511, Vandoeuvre lès Nancy, France. Tel: (+33) 0383157615. Fax: (+33) 0383157660. E-mail: marcelo.decarvalho@univ-lorraine.fr

Supplementary Table S1. Baseline characters of involved studies

| **ID** | **PMID** | **First author** | **Year** | **Features of patients** | | | | **TD** | **Details of techniques** | | | | | | |
| --- | --- | --- | --- | --- | --- | --- | --- | --- | --- | --- | --- | --- | --- | --- | --- |
|  |  |  |  | **Sample size** | **Clinical stage** | **Distribution of carcinoma** | **Negative controls** |  | **Blood sample volume (ml)** | **Sensitivity** | **Specificity** | **Quantification (mean or range)** | **Enrichment** | **Identification** | **Target markers** |
| 21 | 10213205 | Brakenhoff RH | 1999 | 21 | II-IV, R | OC, OP, HP, L | 29 healthy donners | B O | 7 | 2/21=10% | 29/29= 100% | N/S | Erythrocyte lysis + Density gradient separation | RT-PCR | E48 encoding cDNA |
| 22 | 12079157 | Kao RH | 2002 | 7 | III-IV | NP | 15 normal donors | B | 2 | 4/7= 57.14% | 7/15= 46.67% | N/S | Erythrocyte lysis + Density gradient separation | RT-PCR | CK-19+ |
| 23 | 22076949 | Nichols AC | 2012 | 15 | III-IV | OC, OP, HP, L | N/S | B | 7.5 | 6/15=40% | N/S | 0-2/ml | PIMS (Cellsearch) | Cellsearch | EpCAM+CD45- CK8,18, 19+ DAPI+ |
| 24 | 22583603 | Tinhofer I | 2012 | 31 | IVA/B | OC, OP, HP | N/S | B O | 7.5 | 9/31=29% | N/S | N/S | Erythrocyte lysis | Flow cytometry | EpCAM+CK+ CD45- |
| 25 | 22438318 | Hristozova T | 2012 | 33 | III-IV | OP, HP | 20 Normal donors | B | 7.5 | 33.3% | 18/20=90% | 1-2 /3.75ml | NIMS | Flow cytometry | CD45- EpCAM+ CK7/8+ |
| 26 | 23430081 | Bozec A | 2013 | 49 | III-IV | OC, OP | 10 Normal donors | B | 7.5 | 8/49= 16.33% | 10/10= 100% | 0-5 /7.5ml | PIMS (Cellsearch) | CellSearch | EpCAM+CD45- CK8,18, 19+ DAPI+ |
| 27 | 23415697 | Lin HC | 2013 | 28 | III-IV | N/S | 27 normal donors | B P | 4 | 28/28= 100% | 24/27= 88.9% | 109 /ml | NIMS | ICC | CD45-EpCAM+ |
| 28 | 14614011 | Partridge M | 2003 | 40 | I-IV | OC | 16 normal donors | B O P | 7 | 12/40= 30% | 16/16= 100% | N/S | Erythrocyte lysis + Density gradient separation | RT-PCR | E48 encoding cDNA |
| 29 | 21212408 | Wang X | 2011 | 20 | I-IV, R | OC,OP,HP,NP,L | 3 helathy donors | B P | 7.5-15 | 17/19= 89.47% | 3/3=100% | 1-720 /ml | Density gradient separation + Gold nanoparticles | ICC | EGFR+ CK (?)+ |
| 30 | 22682019 | Buglione M | 2012 | 73 | I-III | NP,OP,HP,L,NS,OC | 9 healthy donors | B O | 7.5 | 21/73= 28.77% | 9/9 = 100% | 2 /7.5ml | PIMS (Cellsearch) | Cellsearch | EpCAM+CD45- CK8,18, 19+ DAPI+ |
| 31 | 22844540 | Balasubra-manian P | 2012 | 8 | II-IV | OC,OP | 10 normal donors | B P | 4.5-18.5 | 8/8= 100% | 10/10= 100% | 3 - 3307 /ml | NIMS | ICC | CD45-CK8,18,19+ DAPI+Vimentin+ and EGFR+/ CD44+/ N-cadherin+ |
| 32 | 25105871 | Grisanti S | 2014 | 53 | II-IV | NP,OP,HP,L,NS,OC,CE | 9 normal donors | B O P | 7.5 | 14/53= 26.42% | 9/9= 100% | 5/7.5ml | PIMS (Cellsearch) | Cellsearch | EpCAM+CD45- CK8,18, 19+ DAPI+ |
| 33 | 25057171 | Tinhofer I | 2014 | 144 | I-III | OC, OP, HP, L | N/S | O P | 7.5 | 42/144= 29% | N/S | N/S | NIMS | nested RT-PCR | CD45-EGFR+ |
| 34 | 24844673 | Hsieh JC | 2014 | 53 | II-III | OP,OC,L,HP | 61 healthy donors | O P | 4 | 51/53= 96.22% | 53/61= 86.88% | 75 /ml, no surgery group;19.7/ml, post-surgery | NIMS | RT-PCR | CD45-EpCAM+ podoplanin+ |
| 35 | 18726961 | Yang L | 2009 | 26 | I - III | OC, OP, HP, L | N/S | O | 10-18.5 | 21/26= 80.77% | N/S | 21.7 /ml | NIMS | ICC, RT-PCR | CD45- CK8,18,19+ DAPI+ |
| 36 | 21525401 | Hristozova T | 2011 | 42 | I-IV | OC,OP,HP,NP,L | N/S | B | 7.5 | 18/42=43% | N/S | 1.7 ±0.9 /3.75ml | NIMS | Flow cytometry | CD45-EpCAM+CK+ |
| 37 | 11784252 | Wirtschafter A | 2002 | 18 | I-IV | OC, OP, L, U | N/S | B | 30 | 8/18=44% | N/S | 0-3/30ml | PIMS | ICC | Anti-human epithelial antigen |
| 38 | 12112557 | Lin JC | 2002 | 37 | I-IV | NP | N/S | B | 10 | 83.3% | 64.5% | N/S | Erythrocyte lysis + Density gradient separation | Nested RT-PCR | CK-19+ |
| 39 | 19961621 | Winter SC | 2009 | 16 | I, III, IV | OC,OP,HP,L | N/S | B P | 10 | 11/16= 68.75% | N/S | N/S | PIMS | RT-PCR | EpCAM+ EGFR+ CK19+ELF3+ EphB4+ |
| 40 | 21173379 | Jatana KR | 2010 | 48 | I-IV | OP,OC,L,HP | N/S | B | 10-18 | 71% | N/S | 5 /ml | NIMS | ICC | CD45- CK8,18,19+ DAPI+ |
| 41 | 25479539 | Weller P | 2014 | 10 | I-IV | OP, OC, L | N/S | B P | 20 | 10/10= 100% | N/S | 2-37 / 1000 PBMC | Density gradient separation | ICC | CK+N-cadherin + CD133+ CD45-DAPI+ |
| 42 | 24218516 | Grobe A | 2014 | 80 | I-IV | OC | N/S | O P | 7.5 | 10/80= 12.5% | N/S | 3.2 /7.5ml | PIMS (Cellsearch) | Cellsearch | EpCAM+CD45- CK8,18, 19+ DAPI+ |

**N**= none or not applicable; **N/S** = Non specify; **ID** = serial number of reference; **PMID** = PubMed Unique Identifier; **Clinical stage**: according to American Joint Committee on Cancer (AJCC) cancer staging system, **R**= recurrence; **Distribution of carcinoma**: **OC**, oral cavity; **NP**, nasopharynx; **OP**, oropharynx; **HP**, hypopharynx; **L**, larynx; **NS**, nasal sinus; **U**, unknown primary site; **TD**: Timing of detection (**B** = baseline; **O** = ongoing; **P** = post-treatment); **PIMS** = positive immunomagnetic separation; **NIMS** = negative immunomagnetic separation; **ICC** = immunocytochemistry; **Target markers**: **CK**, cytokeratin; **EGFR**, epidermal growth factor receptor; **ELF3**, E74-Like Factor 3; **EphB4**, Ephrin type-B receptor 4; **DAPI**, 4',6-diamidino-2-phenylindole; **EpCAM**, Epithelial cell adhesion molecule.
